# Evaluating distant recurrence‐free survival and location of metastasis in HER2+ breast cancer by ER status

**DOI:** 10.1002/ijc.70135

**Published:** 2025-09-06

**Authors:** Damien Kaukonen, Alexander Ploner, Erwei Zeng, Jenny Bergqvist, Kamila Czene

**Affiliations:** ^1^ Medical Epidemiology and Biostatistics Karolinska Institutet Stockholm Sweden; ^2^ Department of Surgery and Oncology Capio St Görans Hospital Stockholm Sweden

**Keywords:** breast cancer, flexible parametric modelling, HER2+, location of metastasis, recurrence‐free survival

## Abstract

Prognostic factors, such as the Human Epidermal growth factor Receptor 2 (HER2) and Estrogen Receptor (ER) influence distant recurrence‐free survival (RFS) in breast cancer. This study aims to evaluate the interaction between HER2 and ER status with RFS, and if that interaction influences where the metastasis is located. To do this, we used a study population of all women diagnosed with non‐metastatic, invasive breast cancer in Stockholm from 2007 to 2020. Flexible parametric survival models were used to estimate time‐varying survival and hazard ratios (HR) for RFS. Cumulative incidence was used to quantify rates of metastasis in key locations. We found significant interactions between ER and HER2 for RFS (*p* = 0.037), which was time varying (*p* = 0.017). For ER+ patients, adjusted short‐term survival at 2.5 years after diagnosis was identical for HER2+ compared to HER2− patients (HR 1.02, CI; 0.76–1.39), but was dramatically better for HER2+ patients after 5 years (HR at 7.5 years 0.29, CI; 0.14–0.58). In contrast, among ER− patients, HER2+ patients experienced constant risk compared to HER2− from diagnosis until the end of the study (HR ~0.50). Finally, we observed that HER2+ patients have a higher rate of first metastasis to the brain than HER2− patients (*p* < 0.001). Our study demonstrates that the interaction between ER and HER2 status has a time‐varying impact on RFS and plays a role in determining the location of metastasis. Thus, the utilization of complex models that combine ER and HER2 status can enhance the understanding of patient RFS and the likelihood of metastasis in specific locations.

## What's New?

The prognostic significance of the presence or absence of human epidermal growth factor receptor 2 (HER2) and estrogen receptor (ER) in breast cancer is well‐established. However, the relationship of HER2 and ER with distant disease recurrence and metastasis location is less well understood. This study examined interactions between distant recurrence‐free survival (RFS), metastasis site, and HER2 and ER status. Receptor status was found to interact significantly with RFS in a time‐dependent manner, with combined HER2 and ER status impacting metastasis location. This was most notable for HER2+ cancers, for which increased likelihood of brain metastasis was detected.

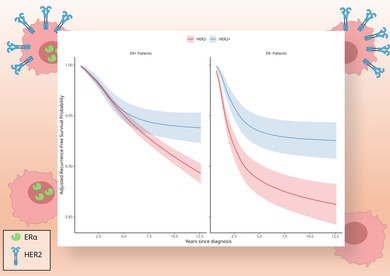

**Abbreviations**
CI: confidence interval
ER: estrogen receptor
FISH: fluorescence in situ hybridisation
HER2: human epidermal growth factor receptor 2
HR: hazard ratio
IHC: immunohistochemistry
NKBC: National Quality Breast Cancer
PR: progesterone receptor
RFS: distant recurrence‐free survival

## 1 BACKGROUND

The relationship between prognostic factors, specifically Human Epidermal growth factor Receptor 2 (HER2) and Estrogen Receptor (ER), and their impact on distant Recurrence‐Free Survival (RFS) remains uncharacterized. Conventionally, breast cancer diagnosis involves evaluating the presence or absence of three receptors: ER, Progesterone Receptor (PR), and HER2. Both the prognosis and treatment approach are contingent upon the presence of each receptor.^1^, ^2^ Among the subtypes, HER2+ and triple‐negative breast cancers have the most unfavorable prognosis.^3^ In the early 2000s, the receptors were classified into five distinct molecular subtypes. This led to their classification as Luminal A and Luminal B (ER+), HER2+ (HER2 subtype), and triple‐negative (basal‐like and claudin‐low).^4^, ^5^, ^6^ The classification of ER+/HER2+ breast cancer as either Luminal B or within the HER2 subtype is conditional upon the differing definitions of each subtype.^4^, ^5^, ^6^ One study revealed that HER2 positivity was only present in 10% of Luminal B tumors, highlighting the influence of other factors on prognosis.^4^, ^5^ Additionally, another study demonstrated the impact of HER2 status on recurrence in Luminal B breast cancers.^4^, ^5^

Previous studies have examined the connection between HER2 status and clinical outcomes,^7^ but the influence of ER status on this association remains unclear. Though breast carcinoma is the most common cancer in women globally, only around 20% of those cases are HER2 over‐amplified (HER2+). Due to HER2 status clinical observation starting only in the early 2000s, long‐term follow‐up for HER2+ patients remains incomplete.^8^, ^9^ Studies have demonstrated the metastasis of HER2 alone or with ER, but only in cases of advanced stage at diagnosis^10^, ^11^ or short follow‐up time.^12^, ^13^, ^14^ While one study had a long follow‐up, its primary focus was on HER2 status and metastasis to the brain.^15^ Consequently, the specific impact of HER2 and ER status on the location of metastasis is yet to be clearly established.

This study seeks to broaden our comprehension of HER2+ and distant RFS by examining the RFS of HER2+ and HER2− patients in the context of ER status. Furthermore, we examined the connection between this and the location of metastasis. With this aim in mind, we have three hypotheses. The combination of HER2 and ER status has a significant impact on RFS. Additionally, the interaction between HER2 and ER changes over time. Lastly, we postulate that the interplay between HER2 and ER status will play a role in determining the location of distant metastasis.

## 2 METHODS

### 2.1 Breast cancer patients

For this study, we used the National Quality Breast Cancer (NKBC) register, the Swedish Cancer register, and the National Cause‐of‐Death register.^16^, ^17^, ^18^ NKBC contains information on all breast cancer patients diagnosed in the Stockholm‐Gotland region from 2007 to 2020. The reports cover a wide range of aspects, including diagnosis, pathology, and follow‐up, as well as local and distant metastasis. The Swedish Cancer register, containing all cancer diagnoses since 1958, supplied us with data on all cancers diagnosed within our cohort. We obtained information from the Cause‐of‐Death register on the cause of death, including death from breast cancer. We began with 20,824 patients diagnosed with their first invasive breast cancer between 2007 and 2020 and had no metastasis at diagnosis. A total of 6222 were excluded from our study because they had been diagnosed with a previous cancer, had unknown HER2 or ER status, were diagnosed at an age older than 75 years, or had <6 months of follow‐up (Figure S1). This gave us a cohort of 14,602 patients, and their characteristics can be found in Table 1.

**TABLE 1:** Baseline patient and tumor characteristics along with treatment for the patient cohort.

	HER2− (*N* = 12,522)	HER2+ (*N* = 2080)	Overall (*N* = 14,602)	*p* value
Age (years)				
50–59	3038 (24.3%)	578 (27.8%)	3616 (24.8%)	<0.001
20–29	75 (0.6%)	27 (1.3%)	102 (0.7%)	
30–39	558 (4.5%)	198 (9.5%)	756 (5.2%)	
40–49	2380 (19.0%)	480 (23.1%)	2860 (19.6%)	
60–69	4505 (36.0%)	602 (28.9%)	5107 (35.0%)	
70–75	1966 (15.7%)	195 (9.4%)	2161 (14.8%)	
ER status				
ER+	11,200 (89.4%)	1407 (67.6%)	12,607 (86.3%)	<0.001
ER−	1322 (10.6%)	673 (32.4%)	1995 (13.7%)	
Tumour grade				
1	2563 (21.1%)	70 (3.7%)	2633 (18.8%)	<0.001
2	6407 (52.8%)	682 (35.9%)	7089 (50.5%)	
3	3157 (26.0%)	1150 (60.5%)	4307 (30.7%)	
Unknown	395	178	573	
Tumour size				
<20 mm	8130 (64.9%)	1140 (54.8%)	9270 (63.5%)	<0.001
20–50 mm	3728 (29.8%)	786 (37.8%)	4514 (30.9%)	
>50 mm	664 (5.3%)	154 (7.4%)	818 (5.6%)	
Lymph node status				
Negative	8642 (70.2%)	1332 (65.6%)	9974 (69.5%)	<0.001
Positive	3677 (29.8%)	698 (34.4%)	4375 (30.5%)	
Unknown	203	50	253	
Chemotherapy				
Adjuvant	4196 (33.8%)	1245 (60.2%)	5441 (37.6%)	<0.001
Neo‐adjuvant	1341 (10.8%)	665 (32.2%)	2006 (13.8%)	
No chemotherapy	6882 (55.4%)	158 (7.6%)	7040 (48.6%)	
Unknown	103	12	115	
Radiation therapy				
Adjuvant	10,552 (85.2%)	1731 (84.4%)	12,283 (85.1%)	0.341
No radiotherapy	1838 (14.8%)	321 (15.6%)	2159 (14.89%)	
Unknown	132	28	160	
Trastuzumab treatment				
Adjuvant	69 (0.6%)	1006 (48.6%)	1075 (7.4%)	<0.001
Neo‐adjuvant	30 (0.2%)	481 (23.3%)	511 (3.5%)	
No Trastuzumab	12,345 (99.2%)	581 (28.1%)	12,926 (89.1%)	
Unknown	78	12	90	
Endocrine treatment				
Adjuvant	10,625 (85.5%)	1349 (65.8%)	11,974 (82.7%)	<0.001
Neo‐adjuvant	188 (1.5%)	16 (0.8%)	204 (1.4%)	
No Therapy	1609 (13.0%)	684 (33.4%)	2293 (15.8%)	
Unknown	100	31	131	
Mode of detection				
Screen detected	5851 (53.1%)	731 (40.7%)	6582 (51.3%)	<0.001
Interval	2479 (22.5%)	510 (28.4%)	2989 (23.3%)	
Clinical	2695 (24.4%)	554 (30.9%)	3249 (25.3%)	
Unknown	625	159	784	

*Note*: Percentages in brackets for HER2+ or HER2− patients. *p* value determined using the chi‐square test, excluding unknown.

Abbreviations: ER, estrogen receptor; HER2, human epidermal growth factor receptor 2.

### 2.2 Variable definitions

We monitored the patients from the time of diagnosis plus 6 months, in order to minimize the influence of metastasis occurring close to the time of diagnosis, until the termination of the study, emigration, local recurrence, distant recurrence, or death. We defined an event as a distant recurrence or death from breast cancer, based on NKBC and National Cause‐of‐Death registry.^16^, ^17^ Censoring occurred with premature end to follow‐up information, such as non‐breast cancer death, local recurrence, or emigration. The NKBC registry provided information for “Brain”, “Lung/Pleura”, “Liver”, “Skeleton”, and “Multiple” metastasis locations.

Patient information was obtained from the NKBC register from 2007 onwards. We stratified age by 10 years, lymph node status was dichotomized into positive or negative, and tumor size was categorized as <20 mm, 20 to 50 mm, or over 50 mm. HER2 and ER statuses were determined from diagnosis and post‐operative pathologist reports. If the information from either report met criteria to be considered positive, then the tumor was considered ER or HER2 positive, respectively. If both reports were missing, then the status was classified as “unknown” The reports included the values for the radioimmunoassay and/or immunohistochemistry (IHC) values for ER status, and the IHC and/or fluorescence in situ hybridization (FISH) for HER2 status. For ER+ the cut‐off values of >10% positive cells for IHC and over 0 fmol/μg DNA for radioimmunoassay, otherwise the status was ER−. For the HER2 status, following the recommended clinical guidelines,^19^ the protein levels from the IHC report were assessed first. If the value reported was 3+, then the tumor was considered HER2+; if the value was <2, then it was HER2−. If the IHC report came back as 2, then the FISH report was used. If the FISH came back positive, then the tumor was considered HER2+; otherwise, the tumor was considered HER2−. Chemotherapy and radiation therapy were both classified as neo‐adjuvant if the patient had received any neo‐adjuvant of their respective therapies; otherwise, they were classified as either adjuvant or no therapy, as reported.

### 2.3 Statistical analysis

We used Kaplan–Meier curves to characterize unadjusted RFS for HER2+ and HER2− patients, both for the total cohort and separately for ER+ and ER− patients. We modeled adjusted RFS via flexible parametric survival models,^20^ including age at diagnosis, tumor grade, tumor size, lymph node status, chemotherapy, and radiation therapy as clinical covariates, with time since breast cancer diagnosis as the time scale. The main model included both main effects and interactions for HER2 and ER status, both fixed baseline effects and time‐varying effects expressed as spline terms. The strategy we used to determine the most suitable degrees of freedom involved the following steps. We began by exploring 21 scenarios, each defined by a combination of seven baseline degrees of freedom (ranging from 2 to 8) and three degrees of freedom for time‐varying effects (2, 3, or 4). Akaike's Information Criterion was used to compare scenarios, with lower scores reflecting a better fit. This resulted in five degrees of freedom allocated to the baseline and three to the time‐varying coefficients. We visualized the fitted model by plotting direct adjusted survival curves, which average individual‐level predicted survival probabilities over time,^21^ for HER2+ and HER2− status, again both for the total cohort and separately for ER+ and ER− patients. Statistical significance of the interaction between HER2 and ER status was assessed via likelihood ratio tests, both overall and separately for time‐constant and time‐varying interaction terms. Sensitivity analyses were conducted separately for the following conditions: all women regardless of age at diagnosis; HER2+ women receiving trastuzumab compared to all HER2− patients; HER2+ versus HER2− in ER+ women treated with either tamoxifen or aromatase inhibitors; and women aged 30–75 at diagnosis. The analysis on HER2+ women receiving trastuzumab was limited to 7.5 years of follow‐up.

We interpreted metastasis location as a multi‐state outcome with five mutually exclusive outcomes of interest, namely a first diagnosis of metastasis specifically to brain, lung, liver, or skeleton, or a first diagnosis of metastasis to multiple locations. We present unadjusted cumulative incidences for these outcomes by HER2 status, again both for the total cohort and separately for ER+ and ER− patients; statistical significance of differences in cumulative incidence between HER2+ and HER2− was assessed via Gray's test.^22^ All the work was done using R version 4.2.0.

## 3 RESULTS

**FIGURE 1:**
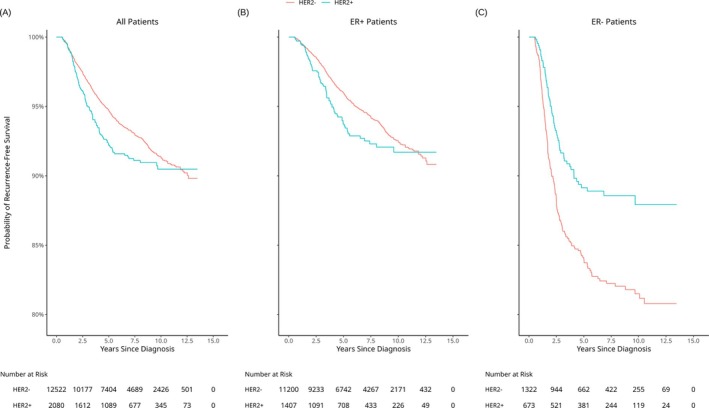
Kaplan–Meier plots for distant recurrence‐free survival, with the log‐rank *p*‐value shown in the bottom left corner. Each plot compares HER2+ patients with HER2− patients among all patients (a), ER+ patients (b), and ER− patients (c). The corresponding risk tables are below their respective plots.

HER2+ patients made up 14.25% (*n* = 2080) of our total number of patients, and they had significantly worse (*p* = 0.02) RFS when compared to the HER2− patients (Figure 1). In the ER+ subgroup, HER2+ patients had worse RFS (*p* = 0.05). In all patients and among the ER+ subgroup, the HER2− patients had a steady rate of RFS. Among the ER− patients, the HER2+ had significantly better RFS for all of follow‐up (*p* < 0.001).

**FIGURE 2:**
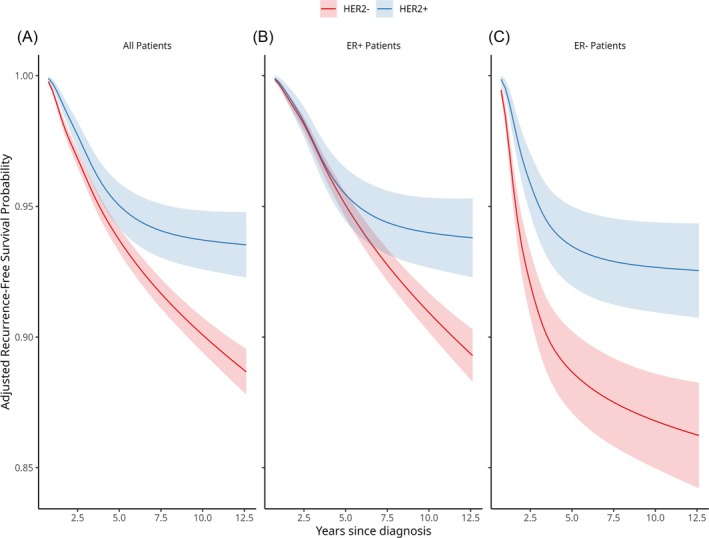
Adjusted recurrence‐free survival curves for HER2+ and HER2− patients among all patients (a), ER+ patients (b), and ER− patients (c), predicted from a flexible parametric survival model for RFS adjusted for age at diagnosis, tumor grade, tumor size, lymph node status, chemotherapy status, and radiation status.

By using flexible parametric models, we found a significant interaction between HER2 and ER status in both our time‐fixed (*p* = 0.037) and time‐varying effect models (*p* = 0.017). In Figure 2, our adjusted RFS probabilities for the three flexible parametric models; one for all patients, one for ER+ patients, and one for ER− patients. After adjusting for covariates, we saw that, overall, HER2+ patients have better long‐term RFS in all cohorts. Among the ER+ patients, RFS is similar within the first 8 years after diagnosis, at which point HER2− has worse survival and continues to have a steady rate of events while the HER2+ curve flattens out. Within the ER− subgroup of patients, we saw that HER2+ has a consistently better RFS from diagnosis until the end of follow‐up.

Evaluating the relative risk of distant recurrence at specific time points (Table 2) for HER2+ compared to HER2−, we saw that HER2+ had a HR of 0.80 (CI 0.61–1.05) at 2.5 years and a HR of 0.54 (CI 0.33–0.88) at 5 years compared to HER2−. HER2+ patients continued to have a further significant reduction in relative risk compared to the HER2− patients for the rest of follow‐up. Within the ER+ group of patients, the HER2+ patients had a similar risk for distant recurrence as the HER2− patients at 2.5 (HR:1.02, CI:0.76–1.39) and were at a significantly reduced risk by 7.5 years (HR: 0.29, CI: 0.14–0.58). The HER2+ patients have a significantly reduced risk of distant recurrence until the end of follow‐up. Last, among the ER− subgroup, we saw that the HER2+ patients were about half as likely to have a distant recurrence compared to the HER2− patients, which maintained a HR around 0.50 for all of follow‐up.

**TABLE 2:** The hazard ratios from our three flexible parametric models for RFS at 2.5, 5, 7.5, and 10 years, using HER2− as baseline for all patients, ER+/HER2− as baseline for the ER+ patients, and ER−/HER2− as baseline for the ER− patients.

Time point	All patients			ER+ patients			ER− patients		
	HR (95% CI)	*p* value		HR (95% CI)	*p* value		HR (95% CI)	*p* value	
2.5 year									
HER2−	1.00 [Reference]			1.00 [Reference]			1.00 [Reference]		
HER2+	0.80 (0.61–1.04)	0.10		1.02 (0.76–1.39)	0.87		0.56 (0.41–0.77)	<0.001	
5.0 year									
HER2−	1.00 [Reference]			1.00 [Reference]			1.00 [Reference]		
HER2+	0.54 (0.33–0.88)	0.01		0.65 (0.45–0.94)	0.02		0.54 (0.39–0.75)	<0.001	
7.5 year									
HER2−	1.00 [Reference]			1.00 [Reference]			1.00 [Reference]		
HER2+	0.22 (0.09–0.53)	0.001		0.29 (0.14–0.58)	<0.001		0.47 (0.32–0.68)	<0.001	
10.0 year									
HER2−	1.00 [Reference]			1.00 [Reference]			1.00 [Reference]		
HER2+	0.09 (0.01–0.78)	0.03		0.14 (0.03–0.68)	0.02		0.42 (0.26–0.69)	<0.001	

*Note*: *p* value determined using Wald test.

Abbreviations: CI, confidence interval; ER, estrogen receptor; HER2, human epidermal growth factor receptor 2; HR, hazard ratio.

The sensitivity analysis for age, which did not exclude patients diagnosed over the age of 74, can be found in Table S1. The analysis which limited HER2+ patients to those who received adjuvant or neo‐adjuvant trastuzumab therapy and their hazard ratios compared to HER2− patients can be found in Table S2. The sensitivity analysis for the different endocrine treatments can be found in Table S3. The results remained unchanged when the study population was limited to women diagnosed between 30 and 75 years of age. All analyses are in line with our primary results.

**FIGURE 3:**
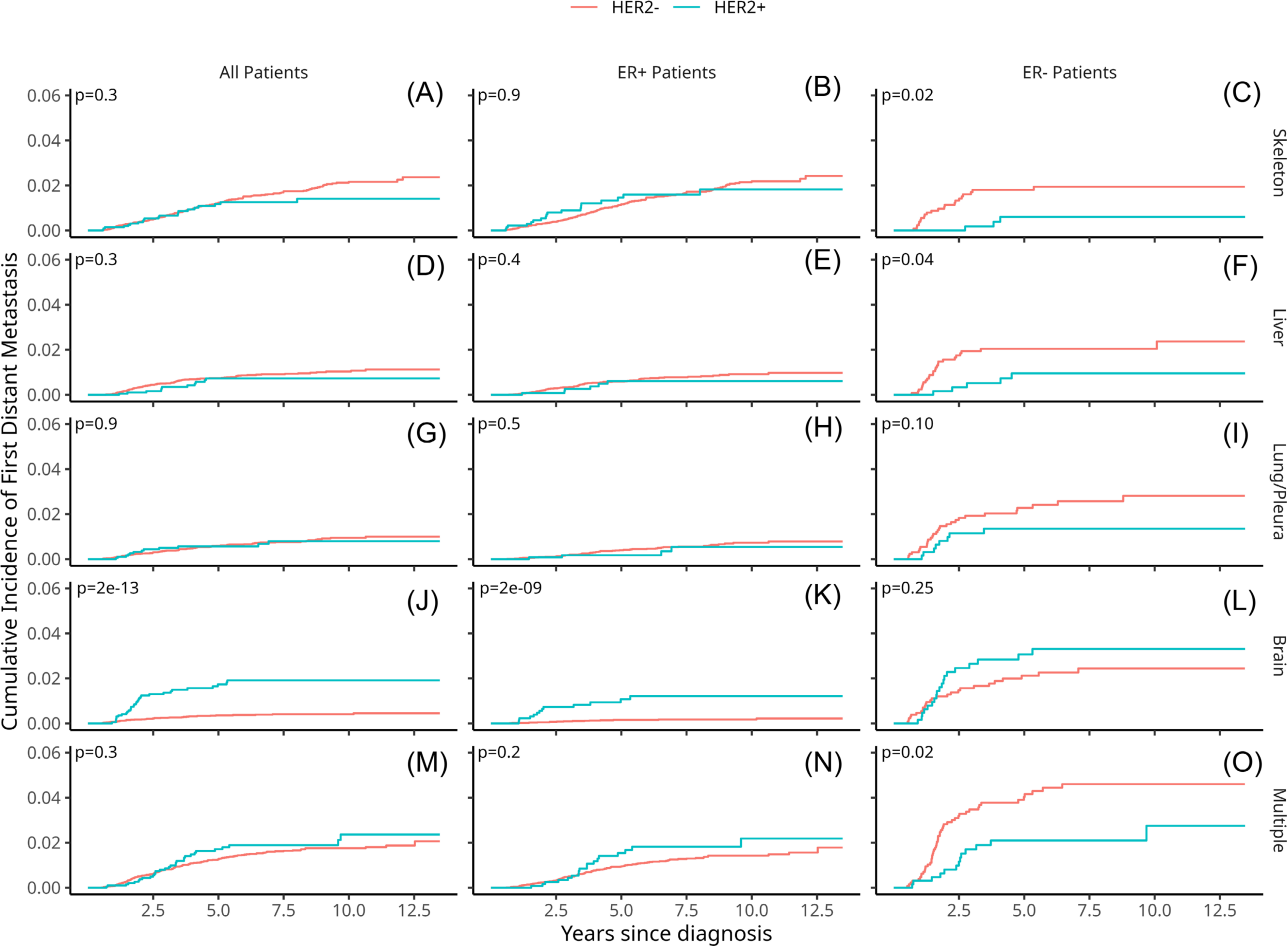
The cumulative incidence plots comparing HER2+ patients with HER2− patients among all (a, d, g, j, m), ER+ (b, e, h, k, n) and ER− (c, f, i, l, o) patients for the first metastasis found in either skeleton (a–c), liver (d–f), lung/pleura (g–i), brain (j–l), or multiple locations (m–o). All locations are mutually exclusive, and the log‐rank *p*‐value for the comparison between HER2+ and HER2− patients within their patient subgroups is shown in the top left corner of their respective plots.

The cumulative incidence rates for the first metastasis to the brain, liver, lung, skeleton, or multiple locations are shown in Figure 3. Among all patients and the ER+ subgroup, we saw that there is no statistically significant difference between HER2+ and HER2− for the skeleton, liver, lung, or multiple locations. In the brain, we saw that HER2+ patients have a significantly higher incidence of first metastasis than HER2− patients in all patients (*p* < 0.001) and the ER+ subgroup (*p* < 0.001). For the ER− patients, HER2− patients have significantly higher rates of first metastasis in the skeleton (*p* = 0.02), liver (*p* = 0.04), and multiple locations (*p* = 0.02) compared to HER2+. Both brain and lung have no significant difference between HER2+ and HER2− among the ER− patients.

## 4 DISCUSSION

In this study, we demonstrated that HER2 and ER status have a combined effect on prognosis. Accounting for tumour characteristics and treatment, we found that there was no significant distinction in prognosis between HER2+ and HER2− among ER+ patients during the initial 5‐year timeframe post diagnosis. On the other hand, within the ER− patient population, HER2+ patients consistently demonstrate a more positive prognosis, both in the short and long term, exhibiting a similar hazard ratio compared to HER2− from the time of diagnosis until the end of the follow‐up period. Furthermore, we found that the combination of HER2 and ER status had an impact on the sites of distant recurrence. Patients with HER2+ breast cancer had a greater likelihood of developing brain metastasis, both overall and within the subgroup of ER+ patients. The contrast between HER2+ and HER2− metastasis was most pronounced in ER− patients, where HER2− showed a higher prevalence of skeletal, liver, and multiple metastases.

The presence of HER2+ is commonly connected to an unfavorable prognosis, and our initial models demonstrate this for both all patients and those with ER+ status.^23^, ^24^ Once tumor characteristics and treatment are factored in, it becomes evident that HER2+ patients have a superior prognosis compared to HER2− patients in all groups. We also found that HER2+ patients exhibited a higher tumor grade, larger tumor size, and positive lymph node status, consistent with previous research.^25^ HER2+ patients are also significantly more likely to have their diagnoses occur between mammographic screenings, known as interval cancer, than HER2− patients. Thus, HER2+ status may be a proxy of a worse disease if these factors, which are all associated with a negative prognosis, are not properly adjusted for. Consequently, what is the reason behind the improved outcomes observed in HER2+ patients? It is our belief that trastuzumab is possibly the most significant factor leading to their improved outcomes in the clinical setting. The results of our sensitivity analysis, which specifically examined HER2+ patients treated with trastuzumab, demonstrated an even more favorable prognosis compared to HER2− patients, thereby further bolstering this hypothesis.

Our observations indicate that HER2+ patients exhibited comparable or lower rates of metastasis compared to HER2− patients in all locations, apart from the brain, consistent with previous findings.^10^, ^11^ The blood–brain barrier prevents trastuzumab from effectively treating brain metastases.^26^ Hence, within a specific bodily region where trastuzumab is absent, it becomes apparent that patients with HER2+ experience a markedly elevated rate of metastases. Other HER2‐targeted drugs used for the treatment of metastatic breast cancer, like lapatinib and more recently and even more efficiently tucatinib^27^ (in combination with trastuzumab and capecitabin) and trastuzumab deruxtecan,^28^ have shown promising treatment results in patients with HER2+ breast cancer and brain metastases. If tucatinib and trastuzumab deruxtecan are available in future treatment of early breast cancer, this may impact both the frequency as well as the location of metastasis. This presents another potential avenue for future research to investigate how the various HER2 treatments influence the interactions observed in this study and how that impacts distant recurrence.

Stratifying patients into the ER+ and ER− subgroups revealed a significant interaction between HER2 and ER status. Moreover, we found that this significant interaction demonstrated a varying effect over time. The short‐term prognosis for both HER2+ and HER2− patients was identical within the ER+ cohort. However, in the ER− cohort, HER2− patients consistently exhibited a more unfavorable prognosis compared to HER2+ patients throughout the entire follow‐up period. In terms of long‐term prognosis, HER2+ patients demonstrated better prognosis regardless of ER status. This underlines the clinical importance of not only personalized diagnostics and personalized medicine, but personalized follow‐up. There is a lot to gain with the right treatment combination from the start based on the ER and HER2 combined status in the adjuvant setting. In conjunction with the findings put forth by others, a comprehensive evaluation of both receptor statuses in combination may lead to improved accuracy in patient prognosis, particularly in the long‐term.^29^, ^30^

It has been previously established that there is crosstalk between the HER2 and ER signaling pathways.^31^, ^32^ This underlying biological phenomenon plays a role in the development of resistance to tamoxifen and aromatase inhibitors.^31^, ^32^ Conducting a study that examines the interactions between HER2 and ER, focusing on the treatments received by ER+ patients, could enhance our comprehension of the survival implications of this crosstalk. The results of our investigation, in conjunction with other relevant studies, have also demonstrated a clear connection between the different receptor combinations and the site of metastasis.^33^, ^34^ This implies that the progression of disease is affected by a biological phenomenon that correlates with receptor status. Additionally, numerous studies have also demonstrated that HER2− primary tumors can undergo a subtype transition and become HER2+ during the process of metastasis, including metastasis to the brain^35^, ^36^ How this impacts our model warrants further investigation. Our research also indicates that we can investigate other prognostic factors that interact within their corresponding biological pathways to improve prognostic modeling.

The investigation of the HER2 and ER status combinations presented several challenges. The adoption of HER2 status in clinical practice has been recent, spanning just over a decade, and HER2+ comprises a small percentage of individuals with breast cancer. As such, we were limited in the follow‐up time and the number of events. The process of further stratifying by ER status results in a further reduction in statistical power, primarily due to the limited representation of ER− patients. All our analyses were limited by the unavailability of data on the specific therapies and regimens received by patients. This includes advancements made in treating HER2+ patients in the past decade, which we could not take into consideration. Despite these circumstances, the differences in RFS and metastasis sites between HER2+ and HER2− breast cancer patients can still be characterized within the framework of ER status. We conducted cross‐referencing of the NKBC, Cancer Registry, and Death Registry utilizing patient ID numbers to obtain comprehensive follow‐up data from a substantial population. Accurate modeling was made possible by incorporating detailed information on metastatic events, metastatic location, tumor characteristics, and treatment regimens.

In summary, our research reveals that the combined influence of HER2 and ER status is time‐dependent and plays a role in determining where distant metastasis occurs. The conclusions drawn from this study indicate that the treatment for HER2 has reached a level of improvement where HER2 positivity alone is not always an indicator of a negative prognosis, particularly in the long term. Additionally, early detection of metastatic events could be facilitated by monitoring specific areas of the body that correspond to the patient's HER2 and ER status. In a broader sense, our results underscore the importance of considering ER status when assessing HER2 status, as it can enhance the predictive value of prognostic models.

## AUTHOR CONTRIBUTIONS

**Damien Kaukonen:** Conceptualization; investigation; writing – original draft; methodology; validation; visualization; writing – review and editing; software; formal analysis. **Alexander Ploner:** Conceptualization; formal analysis; visualization; writing – review and editing. **Erwei Zeng:** Data curation; conceptualization; writing – review and editing. **Jenny Bergqvist:** Writing – review and editing; conceptualization. **Kamila Czene:** Conceptualization; funding acquisition; writing – review and editing; project administration; supervision.

## CONFLICT OF INTEREST STATEMENT

The authors have declared that they have no conflicts of interest.

## ETHICS STATEMENT

The study was approved by the Ethical Committee at Karolinska Institutet (registration number 2019/04369) and was performed in accordance with the Declaration of Helsinki. All patient information contained within this manuscript was anonymized prior to analysis.

## Supporting information


**TABLE S1.** The age sensitivity analysis Hazard ratios for our three groups, All, ER+ and ER− patients, comparing HER2+ to HER2− in each group, and did not exclude patients by age.
**TABLE S2.** The trastuzumab sensitivity analysis Hazard ratios for our three groups, All, ER+ and ER− patients, comparing only HER2+ that received any form of trastuzumab therapy to all HER2− in each group.
**TABLE S3.** The endocrine therapy sensitivity analysis Hazard ratios for ER+ patients, comparing HER2+ to HER2− in women that received tamoxifen or aromatase inhibitors.
**FIGURE S1.** The flowchart of how our patients were selected from NKBC to our cohort used in this study, detailing status at the end of follow‐up for the patients included in the study alongside the information on the number of patients that were excluded from the study.

## Data Availability

Data underlying this research publication were obtained from Statistics Sweden (scb.se) and the National Board of Health and Welfare (Socialstyrelsen, socialstyrelsen.se) and can be directly requested from these institutions. The data in summarized form are available on request to the senior author. Further information is available from the corresponding author upon request.

## References

[1] Abe O , Abe R , Enomoto K , et al. Effects of chemotherapy and hormonal therapy for early breast cancer on recurrence and 15‐year survival: an overview of the randomised trials. Lancet. 2005;365(9472):1687‐1717. doi:10.1016/S0140-6736(05)66544-0 15894097

[2] Slamon D , Eiermann W , Robert N , et al. Adjuvant trastuzumab in HER2‐positive breast cancer. N Engl J Med. 2011;365(14):1273‐1283. doi:10.1056/NEJMoa0910383 21991949 PMC3268553

[3] Baranova A , Krasnoselskyi M , Starikov V , et al. Triple‐negative breast cancer: current treatment strategies and factors of negative prognosis. J Med Life. 2022;15(2):153‐161. doi:10.25122/JML-2021-0108 35419095 PMC8999097

[4] Loi S , Sotiriou C , Haibe‐Kains B , et al. Gene expression profiling identifies activated growth factor signaling in poor prognosis (luminal‐B) estrogen receptor positive breast cancer. BMC Med Genomics. 2009;2(1):1‐9. doi:10.1186/1755-8794-2-37/FIGURES/2 19552798 PMC2706265

[5] Li ZH , Hu PH , Tu JH , Yu NS . Luminal B breast cancer: patterns of recurrence and clinical outcome. Oncotarget. 2016;7(40):65024‐65033. doi:10.18632/ONCOTARGET.11344 27542253 PMC5323135

[6] Tran B , Bedard PL . Luminal‐B breast cancer and novel therapeutic targets. Breast Cancer Res. 2011;13(6):1‐10. doi:10.1186/BCR2904/TABLES/4 PMC3326541 22217398

[7] Bookman MA , Darcy KM , Clarke‐Pearson D , Boothby RA , Horowitz IR . Evaluation of monoclonal humanized anti‐HER2 antibody, trastuzumab, in patients with recurrent or refractory ovarian or primary peritoneal carcinoma with overexpression of HER2: a phase II trial of the gynecologic oncology group. J Clin Oncol. 2003;21(2):283‐290. doi:10.1200/JCO.2003.10.104 12525520

[8] Patel A , Unni N , Peng Y . The changing paradigm for the treatment of HER2‐positive breast cancer. Cancers (Basel). 2020;12(8):1‐17. doi:10.3390/CANCERS12082081 PMC7464074 32731409

[9] Slamon DJ , Clark GM , Wong SG , Levin WJ , Ullrich A , McGuire WL . Human breast cancer: correlation of relapse and survival with amplification of the HER‐2/neu oncogene. Science (1979). 1987;235(4785):182‐191. doi:10.1126/science.3798106 3798106

[10] Huszno J , Nowara E . Risk factors for disease progression in HER2‐positive breast cancer patients based on the location of metastases. Prz Menopauzalny. 2015;14(3):173‐177. doi:10.5114/PM.2015.54341 26528105 PMC4612553

[11] Duchnowska R , Jassem J , Goswami CP , et al. Predicting early brain metastases based on clinicopathological factors and gene expression analysis in advanced HER2‐positive breast cancer patients. J Neurooncol. 2015;122(1):205‐216. doi:10.1007/S11060-014-1704-Y/TABLES/4 25559688 PMC4353882

[12] Arciero CA , Guo Y , Jiang R , et al. ER+/HER2+ breast cancer has different metastatic patterns and better survival than ER−/HER2+ breast cancer. Clin Breast Cancer. 2019;19(4):236‐245. doi:10.1016/J.CLBC.2019.02.001 30846407

[13] Wu SG , Sun JY , Yang LC , et al. Patterns of distant metastasis in Chinese women according to breast cancer subtypes. Oncotarget. 2016;7(30):47975‐47984. doi:10.18632/ONCOTARGET.10099 27322074 PMC5216993

[14] Wu Q , Li J , Zhu S , et al. Breast cancer subtypes predict the preferential site of distant metastases: a SEER based study. Oncotarget. 2017;8(17):27990‐27996. doi:10.18632/ONCOTARGET.15856 28427196 PMC5438624

[15] Guven DC , Kaya MB , Fedai B , et al. HER2‐low breast cancer could be associated with an increased risk of brain metastasis. Int J Clin Oncol. 2022;27(2):332‐339. doi:10.1007/S10147-021-02049-W 34661778

[16] Brooke HL , Talbäck M , Hörnblad J , et al. The Swedish cause of death register. Eur J Epidemiol. 2017;32(9):765‐773. doi:10.1007/S10654-017-0316-1 28983736 PMC5662659

[17] Löfgren L , Eloranta S , Krawiec K , Asterkvist A , Lönnqvist C , Sandelin K . Validation of data quality in the Swedish National Register for breast cancer. BMC Public Health. 2019;19(1):495. doi:10.1186/S12889-019-6846-6 31046737 PMC6498669

[18] Ekbom A . The Swedish multi‐generation register. Methods in Molecular Biology. Vol 675. Humana Press Inc.; 2011:215‐220. doi:10.1007/978-1-59745-423-0_10/COVER 20949391

[19] Thomas J , Loane J , Matthews R , et al. Benchmarking a large regional UK HER2 testing service against current practice guidelines. J Clin Pathol. 2016;70(5):378–382. doi:10.1136/jclinpath-2016-204002 27707772

[20] Liu XR , Pawitan Y , Clements M . Parametric and penalized generalized survival models. Stat Methods Med Res. 2018;27(5):1531‐1546. doi:10.1177/0962280216664760 27587596

[21] Hu ZH , Peter Gale R , Zhang MJ . Direct adjusted survival and cumulative incidence curves for observational studies. Bone Marrow Transplant. 2020;55(3):538‐543. doi:10.1038/S41409-019-0552-Y 31101889 PMC7306148

[22] Kim HT . Cumulative incidence in competing risks data and competing risks regression analysis. Clin Cancer Res. 2007;13(2):559‐565. doi:10.1158/1078-0432.CCR-06-1210 17255278

[23] Perou CM , Sørlie T , Eisen MB , et al. Molecular portraits of human breast tumours. Nature. 2000;406(6797):747‐752. doi:10.1038/35021093 10963602

[24] Koboldt DC , Fulton RS , McLellan MD , et al. Comprehensive molecular portraits of human breast tumours. Nature. 2012;490(7418):61‐70. doi:10.1038/nature11412 23000897 PMC3465532

[25] García Fernández A , Giménez N , Fraile M , et al. Survival and clinicopathological characteristics of breast cancer patient according to different tumour subtypes as determined by hormone receptor and Her2 immunohistochemistry. A single institution survey spanning 1998 to 2010. Breast. 2012;21(3):366‐373. doi:10.1016/J.BREAST.2012.03.004 22487206

[26] Bendell JC , Domchek SM , Burstein HJ , et al. Central nervous system metastases in women who receive trastuzumab‐based therapy for metastatic breast carcinoma. Cancer. 2003;97(12):2972‐2977. doi:10.1002/CNCR.11436 12784331

[27] Lin NU , Borges V , Anders C , et al. Intracranial efficacy and survival with tucatinib plus trastuzumab and capecitabine for previously treated HER2‐positive breast cancer with brain metastases in the HER2CLIMB trial. J Clin Oncol. 2020;38(23):2610‐2619. doi:10.1200/JCO.20.00775 32468955 PMC7403000

[28] Harbeck N , Ciruelos E , Jerusalem G , et al. Trastuzumab deruxtecan in HER2‐positive advanced breast cancer with or without brain metastases: a phase 3b/4 trial. Nat Med. 2024;30(12):3717‐3727. doi:10.1038/s41591-024-03261-7 39271844 PMC11645283

[29] Guan D , Jie Q , Wu Y , Xu Y , Hong W , Meng X . Real‐world data on breast pathologic complete response and disease‐free survival after neoadjuvant chemotherapy for hormone receptor‐positive, human epidermal growth factor receptor‐2‐negative breast cancer: a multicenter, retrospective study in China. World J Surg Oncol. 2022;20(1):326. doi:10.1186/S12957-022-02787-9 36175898 PMC9520808

[30] Lerebours F , Pulido M , Fourme E , et al. Predictive factors of 5‐year relapse‐free survival in HR+/HER2‐ breast cancer patients treated with neoadjuvant endocrine therapy: pooled analysis of two phase 2 trials. Br J Cancer. 2020;122(6):759‐765. doi:10.1038/S41416-020-0733-X 32001832 PMC7078275

[31] Shou J , Massarweh S , Osborne CK , et al. Mechanisms of tamoxifen resistance: increased estrogen receptor‐HER2/neu cross‐talk in ER/HER2‐positive breast cancer. J Natl Cancer Inst. 2004;96(12):926‐935. doi:10.1093/JNCI/DJH166 15199112

[32] Chen Z , Wang Y , Warden C , Chen S . Cross‐talk between ER and HER2 regulates c‐MYC‐mediated glutamine metabolism in aromatase inhibitor resistant breast cancer cells. J Steroid Biochem Mol Biol. 2015;149:118‐127. doi:10.1016/J.JSBMB.2015.02.004 25683269 PMC4380584

[33] Dieci MV , Conte P , Bisagni G , et al. Metastatic site patterns by intrinsic subtype and HER2DX in early HER2‐positive breast cancer. J Natl Cancer Inst. 2024;116(1):69‐80. doi:10.1093/JNCI/DJAD179 37676829 PMC10777675

[34] Darlix A , Louvel G , Fraisse J , et al. Impact of breast cancer molecular subtypes on the incidence, kinetics and prognosis of central nervous system metastases in a large multicentre real‐life cohort. Br J Cancer. 2019;121(12):991‐1000. doi:10.1038/s41416-019-0619-y 31719684 PMC6964671

[35] Priedigkeit N , Hartmaier RJ , Chen Y , et al. Intrinsic subtype switching and acquired ERBB2/HER2 amplifications and mutations in breast cancer Brain metastases. JAMA Oncol. 2017;3(5):666‐671. doi:10.1001/JAMAONCOL.2016.5630 27926948 PMC5508875

[36] Varešlija D , Priedigkeit N , Fagan A , et al. Transcriptome characterization of matched primary breast and Brain metastatic tumors to detect novel actionable targets. J Natl Cancer Inst. 2019;111(4):388‐398. doi:10.1093/JNCI/DJY110 29961873 PMC6449168

