# Supplementary material for: Evaluating distant recurrence‐free survival and location of metastasis in HER2+ breast cancer by ER status
Source: Int J Cancer. 2025 Sep 6;158(5):1184–92. doi: 10.1002/ijc.70135 (PMC12765962; doi:10.1002/ijc.70135)
Supplement: Supplementary file 1 — TABLE S1. The age sensitivity analysis Hazard ratios for our three groups, All, ER+ and ER− patients, comparing HER2+ to HER2− in each group, and did not exclude patients by age. TABLE S2. The trastuzumab sensitivity analysis Hazard ratios for our three groups, All, ER+ and ER− patients, comparing only HER2+ that received any form of trastuzumab therapy to all HER2− in each group. TABLE S3. The endocrine therapy sensitivity analysis Hazard ratios for ER+ patients, comparing HER2+ to HER2− in women that received tamoxifen or aromatase inhibitors. FIGURE S1. The flowchart of how our patients were selected from NKBC to our cohort used in this study, detailing status at the end of follow‐up for the patients included in the study alongside the information on the number of patients that were excluded from the study. [file IJC-158-1184-s001.pdf]

Evaluating Distant Recurrence-Free Survival and Location of Metastasis in HER2+ Breast Cancer by ER status.

Damien Kaukonen, Alexander Ploner, Erwei Zeng, Jenny Bergqvist, Kamila Czene

Table of Contents

Supplementary Tables 2

Supplementary Table 1 2

Supplementary Table 2: 3

Supplementary Table 3 4

Supplementary Figures 5

Supplementary Figure 1 6

Supplementary Tables

Supplementary Table 1: The age sensitivity analysis Hazard ratios for our three groups, All, ER+ and ER- patients, comparing HER2+ to HER2- in each group, and did not exclude patients by age.

	All Patients		ER+ Patients		ER- Patients	
**Time point**	**HR (95%CI)**	**p-value**	**HR (95%CI)**	**p-value**	**HR (95%CI)**	**p-value**
2.5 year						
HER2-	1.00 [Reference]		1.00 [Reference]		1.00 [Reference]	
HER2+	0.86 (0.66-1.12)	0.26	1.11 (0.85-1.45)	0.46	0.57 (0.43-0.75)	< 0.001
5.0 year						
HER2-	1.00 [Reference]		1.00 [Reference]		1.00 [Reference]	
HER2+	0.55 (0.36-0.83)	0.049	0.69 (0.50-0.96)	0.03	0.54 (0.40-0.73)	< 0.001
7.5 year						
HER2-	1.00 [Reference]		1.00 [Reference]		1.00 [Reference]	
HER2+	0.22 (0.09-0.49)	< 0.001	0.31 (0.15-0.61)	< 0.001	0.45 (0.31-0.65)	< 0.001
10.0 year						
HER2-	1.00 [Reference]		1.00 [Reference]		1.00 [Reference]	
HER2+	0.09 (0.01-0.68)	0.02	0.15 (0.03-0.67)	0.01	0.39 (0.24-0.65)	< 0.001

HER2= Human Epidermal growth factor Receptor 2; ER = Estrogen Receptor; HR = Hazard Ratio; CI = Confidence Interval. P-value determined using Wald test.

Supplementary Table 2: The trastuzumab sensitivity analysis Hazard ratios for our three groups, All, ER+ and ER- patients, comparing only HER2+ that received any form of trastuzumab therapy to all HER2- in each group.

	All Patients		ER+ Patients		ER- Patients	
Time point	HR (95%CI)	p-value	HR (95%CI)	p-value	HR (95%CI)	p-value
2.5 year						
HER2-	1.00 [Reference]		1.00 [Reference]		1.00 [Reference]	
HER2+	0.58 (0.41-0.81)	0.001	0.87 (0.62-1.22)	0.41	0.45 (0.30-0.67)	< 0.001
5.0 year						
HER2-	1.00 [Reference]		1.00 [Reference]		1.00 [Reference]	
HER2+	0.20 (0.08-0.51)	< 0.001	0.40 (0.24-0.66)	< 0.001	0.34 (0.22-0.51)	< 0.001
7.5 year						
HER2-	1.00 [Reference]		1.00 [Reference]		1.00 [Reference]	
HER2+	0.03 (0.01-0.31)	0.002	0.10 (0.05-0.20)	< 0.001	0.28 (0.18-0.43)	< 0.001

HER2= Human Epidermal growth factor Receptor 2; ER = Estrogen Receptor; HR = Hazard Ratio; CI = Confidence Interval. P-value determined using Wald test.

Supplementary Table 3: The endocrine therapy sensitivity analysis Hazard ratios for ER+ patients, comparing HER2+ to HER2- in women that received tamoxifen or aromatase inhibitors

	**Tamoxifen**		**Aromatase Inhibitors**	
**Time point**	**HR (95%CI)**	**p-value**	**HR (95%CI)**	**p-value**
2.5 year				
HER2-	1.00 [Reference]		1.00 [Reference]	
HER2+	0.62 (0.29-1.31)	0.208	1.06 (0.66-1.72)	0.803
5.0 year				
HER2-	1.00 [Reference]		1.00 [Reference]	
HER2+	0.36 (0.12-1.10)	0.074	0.61 (0.30-1.26)	0.183
7.5 year				
HER2-	1.00 [Reference]		1.00 [Reference]	
HER2+	0.23 (0.03-1.59)	0.14	0.15 (0.05-0.45)	0.001
10.0 year				
HER2-	1.00 [Reference]		1.00 [Reference]	
HER2+	0.18 (0.01-2.27)	0.185	NA	NA

HER2= Human Epidermal growth factor Receptor 2; ER = Estrogen Receptor; HR = Hazard Ratio; CI = Confidence Interval. P value determined using Wald test.

Supplementary Figures

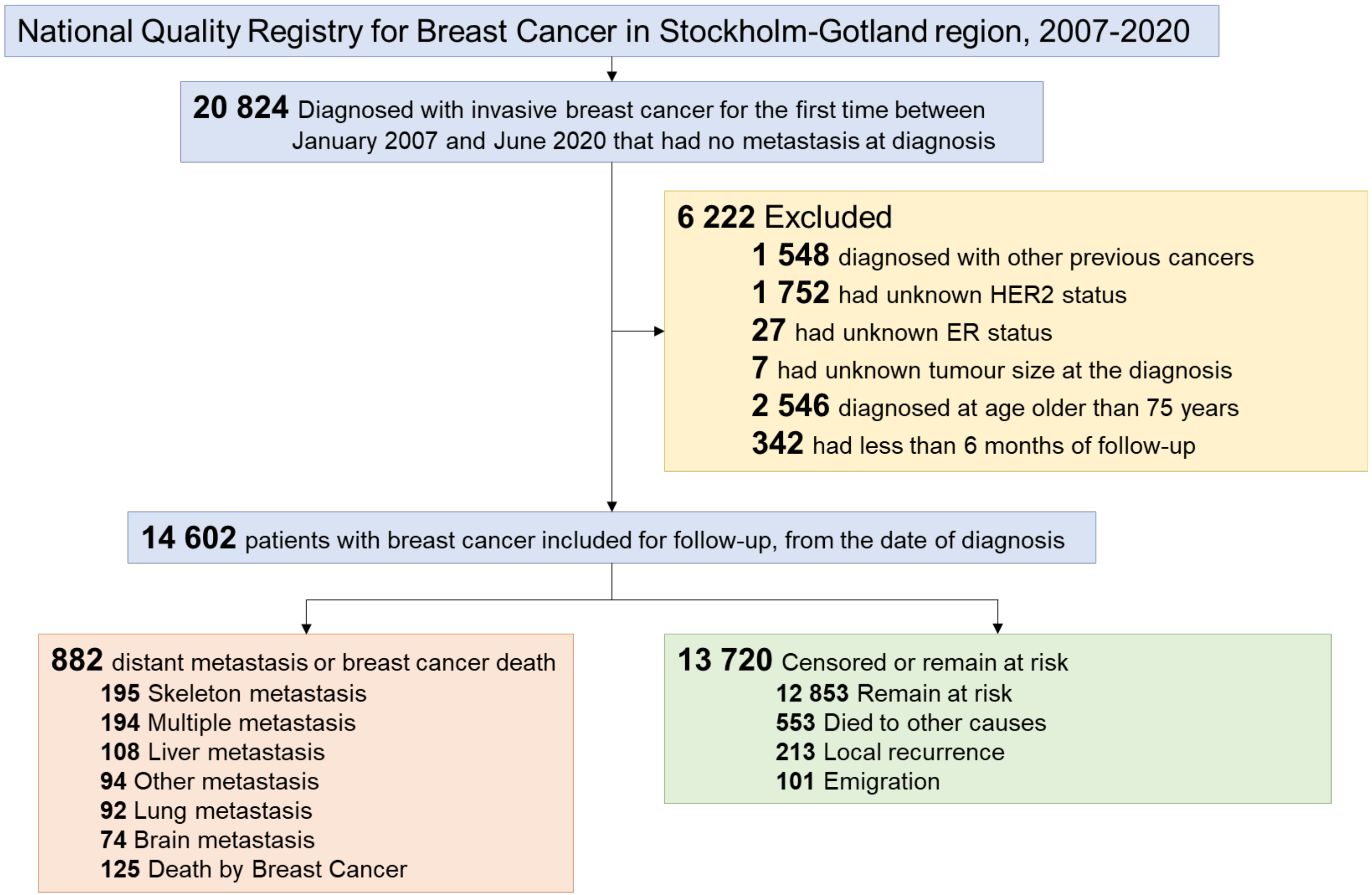

Supplementary Figure 1**:** The flowchart of how our patients were selected from NKBC to our cohort used in this study, detailing status at the end of follow-up for the patients included in the study alongside the information on the number of patients that were excluded from the study
